# Analysis of the Distribution Pattern and Prophage Types in *Candidatus Liberibacter* Asiaticus ‘Cuimi’ Kumquat

**DOI:** 10.3390/plants14010094

**Published:** 2024-12-31

**Authors:** Wen-Ting Li, Xiao-Feng Teng, Li He, Bin Guan, Cui-Ling He, Jian-Jun Liu, Ke-Ling Chen, Zheng Zheng, Jian He

**Affiliations:** 1 National-Local Joint Engineering Laboratory of Citrus Breeding, Cultivation/Horticulture Research Institute, Sichuan Academy of Agricultural Sciences, Chengdu 610066, China; wentingl2015@163.com (W.-T.L.); chen-kl@163.com (K.-L.C.); 2 Agricultural Bureau of Dechang County, Liangshan Yi Autonomous Prefecture, Dechang 615500, China; 3 College of Plant Protection, South China Agricultural University, Guangzhou 510642, China; hcuiling2022@163.com

**Keywords:** Huanglongbing, ‘Cuimi’ kumquat, prophage, distribution pattern

## Abstract

The ‘Cuimi’ kumquat is a unique citrus cultivar known for its thin, crisp pulp and sweet, aromatic flavor. In addition to its use in fresh consumption and processing, this variety exhibits certain medicinal properties. This study aims to investigate the genetic diversity of the Huanglongbing (HLB) bacterium across different tissues of the ‘Cuimi’ kumquat, offering a theoretical basis for understanding the HLB epidemic in Dechang County, Sichuan. The research focuses on the absolute quantification of the HLB bacterium in seven specific tissues of the ‘Cuimi’ kumquat, including new leaves, upper phloem of branches, fruit peduncle, pith, fruit axis, old leaves, and lower phloem of branches. Additionally, the types and contents of prophages were identified in these tissues. In the same diseased branch group, *Candidatus Liberibacter* asiaticus (CLas) exhibited an uneven distribution, with the highest concentration detected in the pith, significantly surpassing levels found in the stem and leaf tissues (new leaves, upper phloem of branches, old leaves, lower phloem of branches). Infected fruit peduncles and pith slices showed noticeable shrinkage and collapse in the phloem. Prophage analysis indicated that multiple types of prophages could be simultaneously detected within the same infected ‘Cuimi’ kumquat branch. New shoot tissues contained both Type 2 and Type 4 prophages, with a relatively higher abundance of Type 4 and a lower abundance of Type 2. The relative abundance of Type 1 prophage in the fruit tissues was generally higher than in other tissues. CLas primarily accumulates in the fruit tissues of the ‘Cuimi’ kumquat, and the situation in Dechang County suggests that individual trees may be infected with multiple prophage strains simultaneously.

## 1. Introduction

Huanglongbing (HLB), caused by the *Candidatus Liberibacter* spp. of the family *Rhizobiaceae* within the class *Alphaproteobacteria*, is the most devastating disease affecting citrus production. In China, HLB is primarily caused by *Candidatus Liberibacter* asiaticus (CLas) [1], posing a significant threat to the citrus industry. Infected plants exhibit symptoms such as mottled and yellowing leaves, mosaic patterns, irregular flushing, excessive and premature flowering, low fruit set, small and deformed fruits, and the persistence of green color at maturity, all of which lead to a severe decline in fruit quality [2]. In recent years, HLB has rapidly spread throughout the Panxi region of China. The origin of the citrus HLB bacterium in this area remains unclear, and a lack of understanding regarding effective control measures has contributed to the disease’s ongoing severity. This has led to weakened tree vigor, degraded fruit quality, and a significant decline in selling prices. The distribution of CLas in infected citrus plants is uneven and varies depending on the variety and the degree of infection [3,4,5,6,7,8]. The present study investigates the distribution patterns of citrus HLB bacteria in different tissues of the ‘Cuimi’ kumquat, as well as the genetic diversity of the strains, providing a theoretical foundation for precise and efficient HLB prevention and control in citrus.

The ‘Cuimi’ kumquat, a new variety bred from a bud mutation of the smooth-skinned kumquat, surpasses other kumquat varieties in both quality and commercial value [9]. In recent years, however, infection by HLB has drastically reduced the yield and quality of the ‘Cuimi’ kumquat, significantly impacting the industry. Some temperate phages can be in the form of plasmids in bacteria or integrate their DNA into the host genome. They become the prophage and are key contributors to bacterial genetic diversity [10]. To date, four types of prophages have been identified in HLB bacteria: Type 1 (SC1), Type 2 (SC2), Type 3 (P-JXGC-3), and Type 4 (CLasMV1) [11,12,13]. Prophage types are commonly used to analyze the population’s genetic structure of CLas [14,15]. Liu et al. (2011) employed the prophage terminase gene as a molecular marker and found significant differences in prophage types between HLB bacteria from Guangdong and Yunnan provinces [16]. Li et al. (2019) identified seven strains with different combinations of prophages among CLas samples collected from eight southern provinces, using prophage typing-specific primers [17]. Zheng et al. (2016) discovered that CLas strains in southern China are primarily of the SC2 prophage type, while those in Yunnan are predominantly of the SC1 prophage type, based on Type 1 and Type 2 prophages [18]. Further, Zheng et al. (2021) revealed substantial population structure differences in HLB bacteria in southern China through whole-genome analysis and amplification using specific primers for three prophage types [19].

In recent years, the ‘Cuimi’ kumquat has been severely impacted by HLB, leading to drastic declines in fruit yield and quality and causing significant losses to the industry. However, research on the symptomatic characteristics of HLB-infected ‘Cuimi’ kumquat branches and fruits, as well as studies on the distribution of the pathogen (CLas), remains limited. Our findings reveal a unique distribution pattern of the prophage within the host tissue, which has important implications for understanding its pathogenesis and transmission mechanisms.

This study utilizes HLB-infected ‘Cuimi’ kumquat branches with fruits from Dechang County as the research material. Through real-time PCR, the distribution of CLas in the vascular tissues (pith) of the infected branches and fruits is analyzed. Optical microscopy is employed to observe the morphological and structural differences between the pith of infected and healthy plants. Additionally, the prophage types of CLas in different tissues are identified. The objective is to uncover the distribution patterns and prophage diversity of CLas in the ‘Cuimi’ kumquat, providing a theoretical foundation for studying HLB epidemiology in Dechang County and the broader Panxi region. Furthermore, our findings suggest potential targets for the development of novel antibacterial agents or genetic approaches to manage HLB.

## 2. Materials and Methods

### 2.1. Experimental Materials

**Figure 1:**
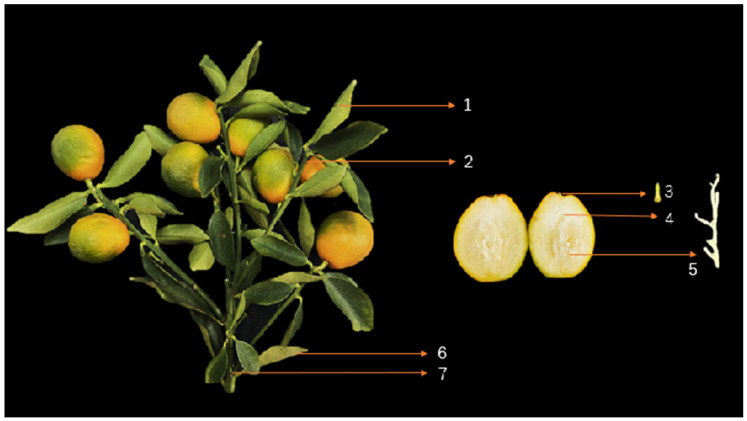
Branches symptoms of ‘Cuimi’ kumquat infected with HLB. Note: 1: New Leaves, 2: Upper phloem of branches, 3: Pedicel, 4: Fruit axis, 5: Pith, 6: Old leaves, 7: Lower phloem of branches.

The branches of ‘Cuimi’ kumquat infected with HLB were collected from Badong Town, Dechang County, Liangshan Prefecture, Sichuan Province, China (102°17′ E, 27°40′ N), in December 2023. Seven branches with fruit displaying HLB symptoms were selected as experimental materials. Each branch was divided, from top to bottom, into the following sections: new leaves, upper phloem of the branch, Pedicel, pith (vascular tissue within the fruit), fruit axis, old leaves, and lower phloem of the branch (Figure 1). Only the midribs of the leaves were taken, and the phloem was collected from the green tissue beneath the bark. All tissues were chopped into small pieces (~2 mm) using a blade. The complete pith was carefully extracted from the stem to the tip of each fruit using tweezers, and three longer sections of pith (≥4 cm) were selected as replicates. These pieces were then cut into three 1.0 cm segments.

### 2.2. Plant DNA Extraction

Each sample was processed individually, using approximately 0.1 g of chopped tissue for DNA extraction. Total DNA was extracted with the HP Plant DNA Kit (200) (2485-02, OMEGA, Atlanta, GA, USA). The concentration and purity of the extracted DNA were measured using a NanoDrop One ultramicro UV-Vis spectrophotometer (Thermo Fisher Scientific, Waltham, MA, USA) and subsequently stored at −20 °C for future use.

### 2.3. Comparison of External Quality Measurements Between Healthy and Infected Fruits

Fifteen healthy and fifteen infected fruits were randomly selected, with three replicates of five fruits each. The transverse and longitudinal diameters of the fruits were measured using a vernier caliper (Deli DL91150, Ningbo, China), and the weight of each individual fruit was determined using an electronic balance (Yousheng ACS-15A, Shanghai, China).

### 2.4. Observation and Measurement of Sections from Healthy and Infected Fruits

The pectin from healthy and infected fruits was selected separately for paraffin section observation and phloem thickness measurement, with three replicates for each group. The fruit pith was divided into two portions using tweezers: one portion was used for DNA extraction and qPCR detection, while the other was initially stored at −20 °C. Based on the qPCR results, appropriate pith samples were selected for paraffin section preparation. The thickness of various tissues in the sections was measured using Image J V1.8.0.112 software. Three slides were examined for each tissue, and ten measurements were taken for the target tissue on each slide. The final thickness value was calculated as the average of these measurements.

### 2.5. Detection and Quantification of CLas

The detection and quantification of CLas were performed using qPCR, following the methodology described by Bao [20]. All DNA samples were tested using the CLas4G/HLBp/HLBr primer-probe combination. A DNA sample known to contain CLas served as the positive control, while a DNA sample from healthy leaves was used as the negative control, and ddH_2_O acted as the blank control. The extracted DNA samples were utilized as templates for qPCR amplification on a Bio-Rad CFX96 (BIO-RAD, Hercules, CA, USA) qPCR instrument. The total reaction mixture volume was 20 μL, consisting of 10 μL of DBI-2041 Bestar qPCR Master Mix (DBI Bioscience, San Diego, CA, USA), 1 μL of DNA template (~25 ng), 0.2 μL of the qPCR probe, 0.4 μL each of forward and reverse primers (10 μmol/L), and 8 μL of ddH_2_O. The amplification protocol was established as follows: an initial denaturation step at 95 °C for 2 min, followed by 40 cycles of denaturation at 95 °C for 10 s, and annealing/extension at 60 °C for 30 s. Data analysis was performed using the default baseline and threshold settings in the Bio-Rad CFX Manager 2.1 software. Upon completion of the program, the Ct values were recorded for further analysis. The presence of CLas in the samples was determined based on the Ct values obtained after the reaction, with a Ct value of ≤30 indicating the presence of the bacterium.

### 2.6. Absolute Quantification of CLas

**Figure 2:**
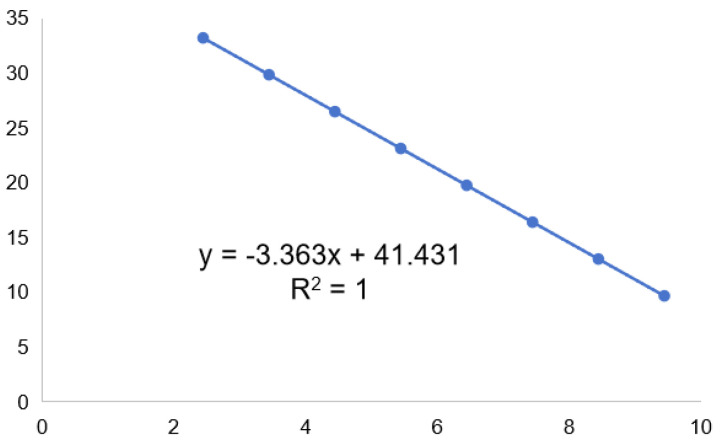
Construction of the standard curves.

A qPCR standard curve was generated using a recombinant plasmid containing the target fragment of the primer in pEASY-T1 (TransGen Biotech, Beijing TransGen Biotech Co., Ltd., Beijing, China). The recombinant plasmid pEASY-T1 (TransGen Biotech., Beijing, China) containing the amplified fragment with primers CLas4G/HLBr/HLBp-Probe was constructed. The initial concentration of the recombinant plasmid using the Qubit 2.0 Fluorometer (Thermo Fisher Scientific, Waltham, MA, USA) was determined, and its copy number was calculated. The standard equation (y = −3.363x + 41.431) (R^2^ = 1) for quantification of CLas was developed (Figure 2). The plasmid copy number was calculated as (Avogadro’s constant × concentration)/(base pair length of the recombinant plasmid × 1 × 10^23^ and the concentration unit is ng/μL. The Ct value of each sample was converted to a copy number using the derived standardization equation. The CLas content in different parts of the same branch was expressed as the number of CLas copies per ng of DNA. The total number of CLas in different segments of the same citrus pectin is calculated by multiplying the number of CLas copies per μL of DNA by the total volume of extracted DNA (60 μL).

### 2.7. Identification of Prophage Types

Seven different parts from infected branches were selected to identify the prophage types in all DNA samples using four prophage type-specific pairs of primers, following the method in [19]. Detailed information regarding the specific primers is provided in Table 1. qPCR was employed for prophage identification, with the reaction conditions and system identical to those described in Section 2.5. A Ct value of ≤30 was considered indicative of the presence of the corresponding type of prophage in the sample. The relative copy number of the prophages was calculated using the 2^−ΔΔCt^ method [21].

### 2.8. Data Processing

Statistical analysis of the experimental data was conducted using SPSS 19.0 and Microsoft Excel 2020. Multiple comparisons were performed using Duncan’s Multiple Range Test (DMRT) within the One-Way ANOVA function of SPSS 26 (IBM Corp., Armonk, NY, USA). Data visualization was carried out using Origin 2018 (OriginLab Corp., Northampton, MA, USA).

## 3. Results and Analysis

### 3.1. Symptoms of ‘Cuimi’ Kumquat Infected with CLas

**Figure 3:**
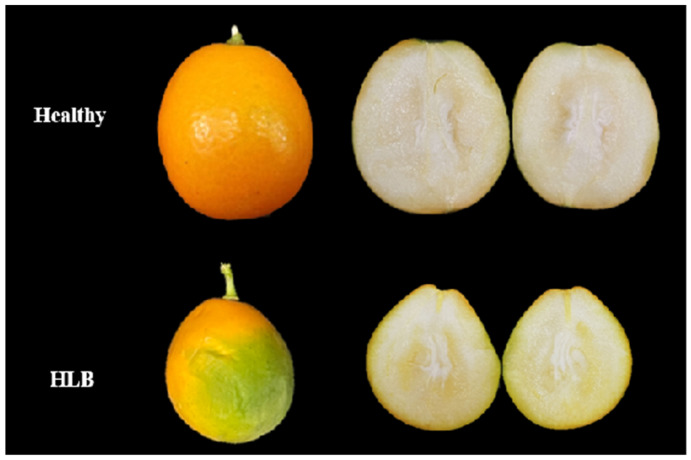
Healthy and HLB-infected ‘Cuimi’ kumquat fruits.

After ‘Cuimi’ kumquat is infected with CLas, the leaves show no obvious symptoms. However, the infected fruits exhibit uneven coloring. The area near the stem turns red while the region near the top remains greenish, which is characteristic of the typical “red nose” fruit symptom. In comparison to healthy fruits, the infected ones vary in size and shape, displaying asymmetrical deformities (Figure 3).

### 3.2. Differences in Appearance Quality Between Healthy and Infected Fruits

Measurements of the external quality between healthy and infected fruits revealed that the average weight of healthy ‘Cuimi’ kumquats was 39.75 g, which is 63.98% heavier than that of the infected fruits and significantly greater (*p* < 0.01). Both the transverse diameter (40.92 mm) and longitudinal diameter (45.95 mm) of healthy fruits were significantly larger than those of the infected fruits, which measured 28.22 mm and 30.68 mm, respectively (*p* < 0.01). These results indicate that infection with CLas significantly reduces the size of ‘Cuimi’ kumquat fruits (Table 2).

### 3.3. Comparison of Tissue Morphology Between the Pectin of Healthy and Infected Fruits

**Figure 4:**
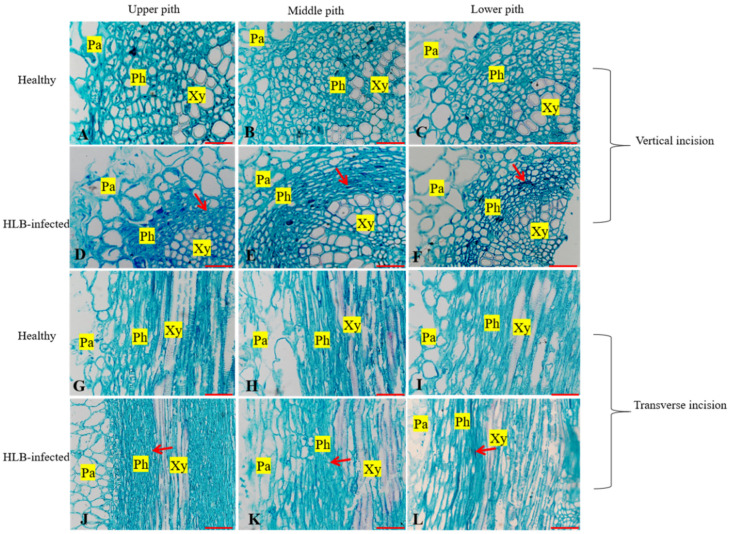
Anatomical structure of pith from healthy and infected fruits. Note: Pa: parenchyma; Ph: phloem; XY: xylem. Red arrows denote wrinkled phloem. Bars = 50 μm. (**A**) The upper section of healthy citrus pith with the horizontally cut; (**B**) The middle section of healthy citrus pith with the horizontally cut; (**C**) The lower section of healthy citrus pithwith the horizontally cut; (**D**) The upper section of HLB-infected citrus pith with the horizontally cut; (**E**) The middle section of HLB-infected citrus pith with the horizontally cut; (**F**) The lower section of HLB-infected citrus pith with the horizontally cut; (**G**) The upper section of healthy citrus pith with the vertically cut; (**H**) The middle section of healthy citrus pith with the vertically cut; (**I**) The lower section of healthy citrus pithwith the vertically cut; (**J**) The upper section of HLB-infected citrus pith with the vertically cut; (**K**) The middle section of HLB-infected citrus pith with the vertically cut; (**L**) The lower section of HLB-infected citrus pith with the vertically cut.

Microscopic examination of the cross-sections and longitudinal sections of the pith revealed deformation and shrinkage in the upper, middle, and lower sections of the pith in infected fruits. Slight deformation was observed in the upper part of the pith, accompanied by significant thickening of the phloem upon longitudinal sectioning (Figure 4D,J). In the middle section of the pith, the phloem was compressed and deformed, leading to the deformation and rupture of cell walls (Figure 4E,K). The lower section of the pith exhibited thickening and deformation, along with shrinkage of the phloem (Figure 4F,L). Comparative measurements indicated that the phloem thicknesses in the upper, middle, and lower parts of infected fruits were 90.45%, 89.75%, and 88.15%, respectively, of those in the corresponding healthy fruits (Table 3).

### 3.4. Detection Results of CLas in Branches with Fruits of ‘Cuimi’ Kumquat

The ‘Cuimi’ kumquats used in this study were collected during the fruit ripening period in December. qPCR was employed to test 40 suspected diseased ‘Cuimi’ kumquat samples. The positive rate for CLas detected by qPCR was 98%. Subsequently, seven branches exhibiting typical symptoms were selected for part-specific detection. The results indicated that CLas could be stably detected in the fruit pith, fruit stem, and central axis of all infected branches, while some samples from other parts did not test positive for CLas (Table 4).

### 3.5. Distribution of CLas in Different Parts of ‘Cuimi’ Kumquat Branches

qPCR detection revealed an uneven distribution of CLas among various tissues. The CLas content in fruits (pith, central axis, and fruit stem) was significantly higher than in other tissues (*p* < 0.05). In contrast, the CLas content in the phloem of new shoots, new leaves, old leaves, and fruit-bearing branches was relatively low (Table 4). Specifically, the CLas content in fruits, from highest to lowest, was as follows: pith (7.36 ± 5.83 copies/ng DNA) > central axis (6.76 ± 5.37 copies/ng DNA) > fruit stem (6.19 ±4.96 copies/ng DNA). Among other tissues, the CLas content, from highest to lowest, was as follows: upper phloem of branches (3.28 ± 2.80 copies/ng DNA) > lower phloem of branches (2.93 ± 2.35 copies/ng DNA) > new leaves (2.50 ± 2.20 copies/ng DNA) > old leaves (2.44 ± 1.75 copies/ng DNA).

### 3.6. Analysis of Prophage Types of CLas in ‘Cuimi’ Kumquat

Real-time fluorescence PCR results showed that multiple types of prophages were detected simultaneously in the same infected ‘Cuimi’ kumquat branches. In the seven selected branch samples, Type 1 prophage was detected individually in new leaves, fruit peduncles, old leaves, and the phloem of the lower branches, and both Type 2 and Type 4 prophages were detected simultaneously. Type 1, Type 2, and Type 4 prophages were detected in the pedicel and fruit axis. In the old leaf section, Type 1 prophage was detected alone, as well as combinations of Type 2 and Type 4 prophages. In the upper phloem of the branches, Type 1 prophage was detected individually, along with combinations of Type 2 and Type 4, and Type 1, Type 2, and Type 4 prophages (Table 5). These findings indicate that four types of CLas strains exist within the same infected ‘Cuimi’ kumquat, with combinations of Type 2 and Type 4 prophages present in all parts of the branches. Among all branch sections, the relative abundance of Type 1 and Type 4 prophages was higher, while the relative abundance of the Type 2 prophage was lower (Table 5). The relative abundance of the Type 1 prophage in fruit tissues was generally higher than in other tissues (Table 5).

### 3.7. Distribution Pattern of CLas and Prophage in the Pith of ‘Cuimi’ Kumquat Fruits

**Figure 5:**
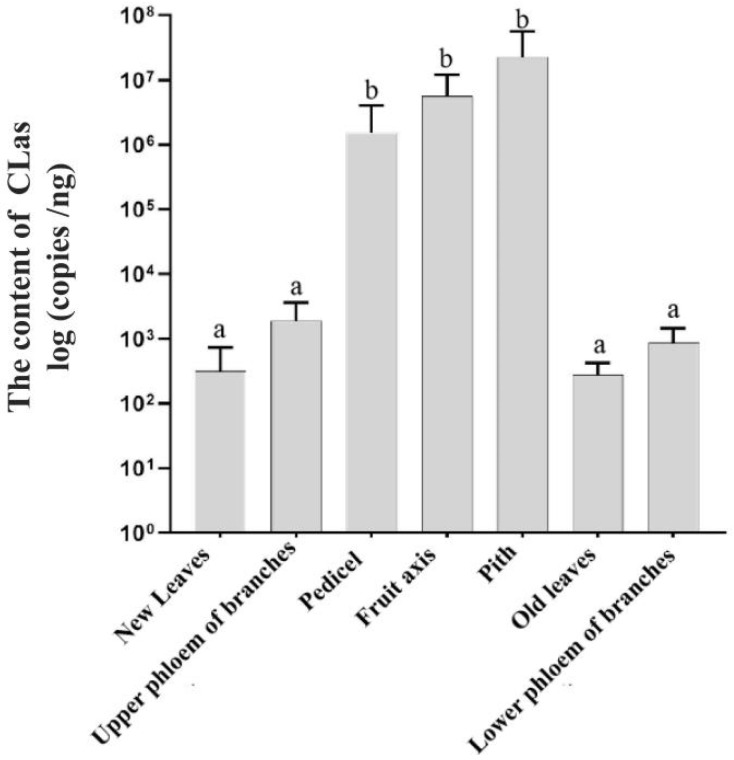
The content of CLas in different tissues of ‘Cuimi’ kumquat. Note: different letters indicate significant differences in 95% confidence intervals (Duncan’s, *p* < 0.05).

**Figure 6:**
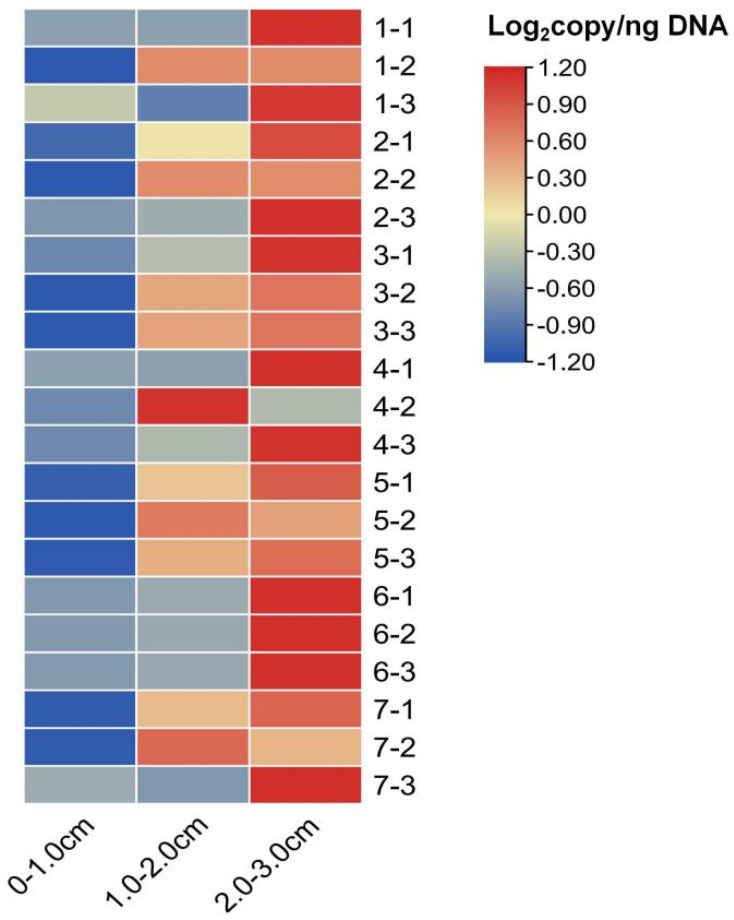
Content of CLas in different segments of ‘Cuimi’ kumquat peduncles. Note: Different patterns represent the copy number of CLas: 0–1 cm represents the upper pith, 1.0–2.0 cm represents the middle pith, 2.0–3.0 cm represents the lower pith. The numbers on the right represent the sample numbers.

qPCR quantitative analysis revealed that the distribution of CLas within the same fruit was uneven. Specifically, there was no significant difference (*p* < 0.05) in the average CLas content between the upper segment (0–1.0 cm) of the orange pith and the middle-to-lower segments. The highest average CLas content was found in the segment located between 2.0 and 3.0 cm (7.45 ± 4.82 copies/ng DNA), which was significantly higher than that in the other two segments (*p* < 0.05) (Figure 5 and Figure 6). Analysis of different orange pith samples revealed that the uneven distribution of CLas within the pith did not follow a clear pattern. However, the results indicated that CLas was predominantly concentrated in the 1.0–2.0 cm and 2.0–3.0 cm segments, which represent the middle part of the fruit. Similarly, the distribution of the Type 1 phage in the orange pith was also uneven, with higher levels observed in the 1.0–2.0 cm segment. Unlike the pathogen content, the distribution of the Type 1 phage was not confined to a specific area. Rather, it was also concentrated in both the 1.0–2.0 cm and 2.0–3.0 cm segments of the pith.

## 4. Discussion

The cultivation of ‘Cuimi’ kumquat is a significant economic source for farmers in Dechang County. However, in recent years, it has been severely impacted by CLas, and there is a lack of detailed descriptions of symptoms and pathogen research related to CLas in ‘Cuimi’ kumquat. Through observation, the authors found that infection with CLas leads to symptoms such as leathery and thickened leaves, deformed and asymmetrical fruits, and aborted seeds, resembling the symptoms seen in CLas-infected Gonggan citrus [4]. Studies on the distribution of CLas in infected ‘Cuimi’ kumquats revealed that its content is significantly higher in fruit tissues, including the fruit stem, pith, and central axis, which aligns with the distribution pattern of CLas in infected ‘Shatangju’ branches [21]. This study demonstrated that the distribution of CLas in various parts of the kumquat is uneven, with a pronounced enrichment in fruits and surrounding tissues (sink organs), similar to findings in other citrus varieties [21,22,23,24].

Tatineni et al. (2008) observed through electron microscopy that CLas can move through sieve plates in plants, speculating that its movement direction aligns with the flow of nutrients—from source organs (old leaves) to sink organs (new leaves, fruits, flowers, etc.) that store or consume nutrients [6]. This phenomenon helps explain the observed enrichment of CLas in fruits across many studies [6]. Furthermore, the high concentration of CLas in the fruit pith may stem from the abundant vascular bundle tissue, composed of both phloem and xylem, found in the pith. These vascular bundles transport essential nutrients for fruit development and connect to the fruit pulp, creating favorable nutritional conditions and sites for the growth of CLas [25]. Research shows that utilized electron microscopy to observe that the sieve tube cells in the phloem of CLas-infected fruit stalks exhibited irregular arrangements, with substantial starch accumulation leading to sieve tube blockage [24]. They hypothesized that this blockage may impede the movement of CLas, contributing to its uneven distribution [24]. Similarly, sing transmission electron microscopy, found a high abundance of CLas in the phloem cells of the fruit pith, while adjacent phloem cells contained significantly less or even no CLas [25].

This observation provides an anatomical explanation for the uneven distribution of CLas within the fruit pith [25]. Previous studies have indicated that among CLas strains, the Type 1 strain often coexists with other bacteriophage types [19]. However, this study found that only the single Type 1 prophage was detected in various parts of the ‘Cuimi’ kumquat, suggesting that this prophage may represent the inherent CLas strain type, or the dominant strain, in this region. In other tissues of ‘Cuimi’ kumquat, a combination of Type 2 and Type 4 prophages was identified, indicating potential cross-infection within the same branch. The transmission of CLas primarily depends on the insect vector, the citrus psyllid, and Type 2 CLas strains are more likely to proliferate in adult citrus psyllids, suggesting that Type 2 strains are highly infectious [26]. This finding implies that the Type 2 and Type 4 strains observed in the Dechang region may result from foreign invasion. In recent years, the citrus industry in Sichuan has experienced rapid development, with the introduction and planting of many new varieties, which may account for the emergence of multiple CLas populations in Dechang County.

## 5. Conclusions

Analysis of the distribution pattern of HLB in ‘Cuimi’ kumquat revealed that CLas is enriched in fruit tissues and unevenly distributed among branches bearing fruit. Further investigation indicated that the same fruit branch harbors CLas strains with diverse prophage types. The Type 1 prophage represents the inherent CLas population, while the Type 2 and Type 4 prophages constitute the invading CLas population. The CLas strains in the Panxi region of Sichuan Province exhibit significant polymorphism. Conducting relevant prophage diversity analyses will provide insights into the molecular mechanisms underlying local CLas population variation, thereby contributing valuable information to the genetic diversity and epidemiological studies of CLas populations in China.

## Figures and Tables

**Table 1:** Real-time PCR primer information for detection of CLas and their prophage types.

Type	Primer Name	Sequences (5′–3′)	Length (bp)	Genes
CLas	CLas_4G	AGTCGAGCGCGTATGCGAAT	78	16S rRNA [20]
	HLBr	GCGTTATCCCGTAGAAAAAGGTAG		
	HLBp-Probe	FAM-AGACGGGTGAGTAACGCG-BHQ1		
Type 1	SC1-045F	CCGTTCGTCTTTTGCCCATA	87	SC1_gp045 [19]
	SC1-045R	GCATTCTTCGCATCATCGGA		
Type 2	SC2-035F	AGGTCACAAGGATTTAGCCCA	86	SC2_gp040 [19]
	SC2-035R	CTCCTAATCCCGCACCGATA		
Type 3	PJXGC-8F	CGGCGCTGAACTCTTGTATT	85	PJXGC_08 [12]
	PJXGC-8R	AAGGGCGTTGTTCTTGTCAC		
Type 4	MV1-1F	ACGACCACATGACCAGACTT	93	CLasMV1_ORF3 [21]
	MV1-1R	TGATGCGTATAAGGAGTTGACTG		

**Table 2:** Comparison of appearance quality between healthy and CLas-infected fruits.

	Measurement Indicators			
	Single Fruit Weight (g)	Transverse Diameter (mm)	Longitudinal Diameter (mm)	Fruit Shape Index
Healthy Fruit	39.75 ± 0.31 a	40.92 ± 0.18 a	45.95 ± 0.29 a	1.12 ± 0.01 a
CLas-infected fruit	24.24 ± 0.42 b	28.22 ± 0.11 b	30.68 ± 0.18 b	1.08 ± 0.01 b

Note: Different lowercase letters in the same column represent significant differences at a 1% level.

**Table 3:** Measurement and comparison of the phloem thickness of disease-infected and healthy kumquat.

Kumquat on Top		Kumquat in the Middle		The Lower Part of Kumquat	
Phloem Thickness Ratio	*p*-Value	Phloem Thickness Ratio	*p*-Value	Phloem Thickness Ratio	*p*-Value
90.45%	0.09	89.75%	0.07	88.15%	0.05

Note: Thickness ratio of the phloem: The thickness ratio of the phloem in infected citrus pectin compared to that in healthy citrus pectin.

**Table 4:** Detection of CLas in ‘Cuimi’ kumquat samples by qPCR.

No.	New Leaves	Upper Phloem of Branches	Fruit Pedicel	Fruit Pith	Fruit Axis	Old Leaves	Lower Phloem of Branches
1	33.52	22.04	18.78	17.23	18.12	33.56	23.66
2	28.73	33.21	22.05	17.21	17.72	27.19	25.56
3	31.01	27.97	21.76	19.78	20.78	29.65	30.15
4	28.41	29.98	23.35	20.23	22.35	28.16	29.58
5	29.12	33.56	18.93	15.73	18.92	31.44	32.54
6	28.12	28.54	24.38	22.91	21.28	30.64	32.55
7	28.01	29.29	16.34	14.68	18.19	27.15	28.25

Note: The number represents the branch number, and the value represents the Cycle threshold (Ct) value. A value greater than 30 is considered negative, and a value less than 30 is considered positive.

**Table 5:** The types and relative abundances of prophage of CLas in different tissues of ‘Cuimi’ kumquat.

No.	New Leaves	Upper Phloem of Branches	Fruit Pedicel	Fruit Pith	Fruit Axis	Old Leaves	Lower Phloem of Branches
1	1.03 (T1)	0.58 (T1)	1.41 (T1)	17.68 (T1)	1.45 (T4)	1.08 (T1)	0.88 (T1)
2	2.65 (T1)	0.44 (T1)	3.48 (T1)	1.70 (T1)	1.41 (T1)	1.22 (T1)	0.29 (T1)
3	0.51 (T2)	0.36 (T2)	0.94 (T4)	0.69 (T2)	0.89 (T2)	0.73 (T2)	0.62 (T2)
	0.62 (T4)	0.83 (T4)		2.76 (T4)	0.48 (T4)	0.25 (T4)	0.72 (T4)
4	0.42 (T2)	0.55 (T2)	0.42 (T2)	0.75 (T2)	0.43 (T2)	0.31 (T2)	0.42 (T2)
	0.80 (T4)	1.64 (T4)	0.63 (T4)	4.85 (T4)	0.73 (T4)	0.93 (T4)	0.55 (T4)
5	0.77 (T1)	0.64 (T1)	0.79 (T1)	2.60 (T2)	4.59 (T1)	1.15 (T1)	0.85 (T1)
		0.58 (T2)		1.21 (T4)			
		0.97 (T4)					
6	0.30 (T2)	0.52 (T1)	4.0 (T4)	3.29 (T2)	0.44 (T2)	0.40 (T2)	0.24 (T2)
		0.50 (T2)		0.41 (T4)	1.81 (T4)	0.41 (T4)	0.65 (T4)
	1.61 (T4)	0.06 (T4)					
7	0.27 (T2)	1.67 (T2)	1.80 (T4)	2.25 (T2)	0.51 (T2)	0.38 (T2)	1.25 (T2)
	0.43 (T4)	0.89 (T4)		17.23 (T4)	0.30 (T4)	0.36 (T4)	0.57 (T4)

## Data Availability

All publicly published data in this study are available upon request from the corresponding author.
